# Analysis of the miR-34 family functions in breast cancer reveals annotation error of miR-34b

**DOI:** 10.1038/s41598-017-10189-1

**Published:** 2017-08-28

**Authors:** M. E. Engkvist, E. W. Stratford, S. Lorenz, L. A. Meza-Zepeda, O. Myklebost, E. Munthe

**Affiliations:** 1 Department of Tumour Biology, Institute for Cancer Research, Oslo University Hospital, Oslo, Norway; 2 Department of Core Facilities, Institute for Cancer Research, Oslo University Hospital, Oslo, Norway; 3 Department of Clinical Science, University of Bergen, Bergen, Norway

## Abstract

The microRNAs in the miR-34 family, consisting of miR-34a, miR-34b and miR-34c, are tumour suppressors. The annotated human miR-34b-5p has one additional base at the 5’ end of the common miR-34 family seed sequence, compared to miR-34a-5p and miR-34c-5p. This extra base results in a shift of the seed sequence, which would affect the target gene repertoire and have functional consequences. During our studies of miR-34 functions, we investigated the precise sequence of mature miR-34b-5p in human cells by deep sequencing. We found that a miR-34b-5p without the extra base was the predominant form in both non-malignant and malignant cells derived from several human tissues, indicating that the miR-34b annotation is misleading. We evaluated the functional implications of the seed shift, by comparing the effect of mimics representing the alternative miR-34b-5p sequences in MDA-MB-231 cells. In contrast to the annotated miR-34b, the endogenously expressed miR-34b displayed tumour suppressive characteristics *in vitro* similarly to miR-34c. These data demonstrate the importance of determining the precise sequence of a mature microRNA before exploring miRNA functions.

## Introduction

MicroRNAs (miRNAs) are small, non-coding RNAs that affect many fundamental biological processes, such as development, cell differentiation and cell growth, by functioning as regulators of gene expression. One miRNA generally regulates many genes and deregulation of miRNAs is often associated with human diseases, including cancer^1^. After transcription, miRNAs go through a stepwise maturation process including progressive cleaving, resulting in a cytoplasmic RNA duplex. Successively, the RNA duplex is loaded into the RNA-induced silencing complex (RISC), where one of the two miRNA arms is incorporated as the mature miRNA guide. The asymmetric selection is a non-random process where the strand with the least thermodynamically stable 5′ terminus is preferred^2^. The miRNA originating from the forward strand of the duplex is named 5p, while the miRNA originating from the reverse strand is named 3p. As part of the RISC, the mature miRNA binds target mRNAs, leading to reduced protein production through degradation or translational repression of the mRNA^3, 4^. For gene silencing to occur, the target mRNA must be complementary to the miRNA seed sequence, commonly defined as nucleotide 2–7^5^.

MiRBase is the most used miRNA database annotating miRNAs, and is widely used by the scientific community and by commercial companies that develop tools to study miRNAs, like synthetic mimics, primers and data programs predicting binding sites. However, there is increasing evidence that there can be variation in the termini of the mature miRNA sequences. The most predominantly expressed sequence of a specific miRNA is annotated as the mature miRNA, also referred to as the canonical or the reference miRNA, while less expressed sequences are referred to as isomiRs^6, 7^.

MiRNAs with conserved seed sequences are grouped into miRNA families. The consensus is that members of the same miRNA family target a related set of genes, and thus are to some extent biologically redundant, but may allow multiple regulatory mechanisms and expression profiles in different cells or conditions. The human miR-34 family consists of three members, miR-34a, miR-34b, and miR-34c. The miR-34 miRNAs are tumour suppressors and are critical mediators in the p53 pathway^8, 9^. In particular, it has been shown that the miR-34 family members reduce cell growth, induce apoptosis and affect cell migration^10, 11^. Loss of miR-34 is strongly associated with cancer and miR-34 replacement therapy is currently in clinical trials for treatment of primary liver cancer and other selected cancer types with liver metastasis^12^. Mir-34a is encoded by its own gene located in chromosome segment 1p36. MiR-34b and miR-34c are encoded from the same locus situated on chromosome 11q23, and expressed as a bicistronic transcript. In humans miR-34b-5p has an additional base at the 5′ end, shifting its seed sequence by one base, relative to the other miR-34 family members as annotated in databases like miRBase and miRNAMap 2.0 and found in scientific reviews^13–15^.

To identify the common and unique effects of the bicistronic miR-34b and miR-34c we introduced miR-34b and miR-34c mimics into the breast cancer cell line MDA-MB-231. This cell line is derived from a highly aggressive metastatic breast cancer with low levels of endogenous miR-34. The global transcript levels and tumour suppressive characteristics varied greatly between the two mimics. Sequencing of miR-34b in these cells demonstrated that the endogenous miR-34b did not match the annotated miR-34b. Furthermore this was confirmed in other datasets. Functional analyses demonstrated that the miR-34b expressed in the MDA-MB-231 cells had tumour suppressive capacity resembling that of miR-34c, while the annotated miR-34b did not.

## Results

### MiR-34b-5p and miR-34c-5p exert different functions in MDA-MB-231 cells

**Figure 1:**
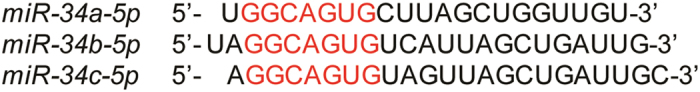
Mature human miR-34 family sequence. Sequence alignment of the mature miR-34a-5p, miR-34b-5p and miR-34c-5p molecules, as annotated by miRBase, the primary repository for miRNA sequences. The miR-34 family seed sequence is highlighted in red.

**Figure 2:**
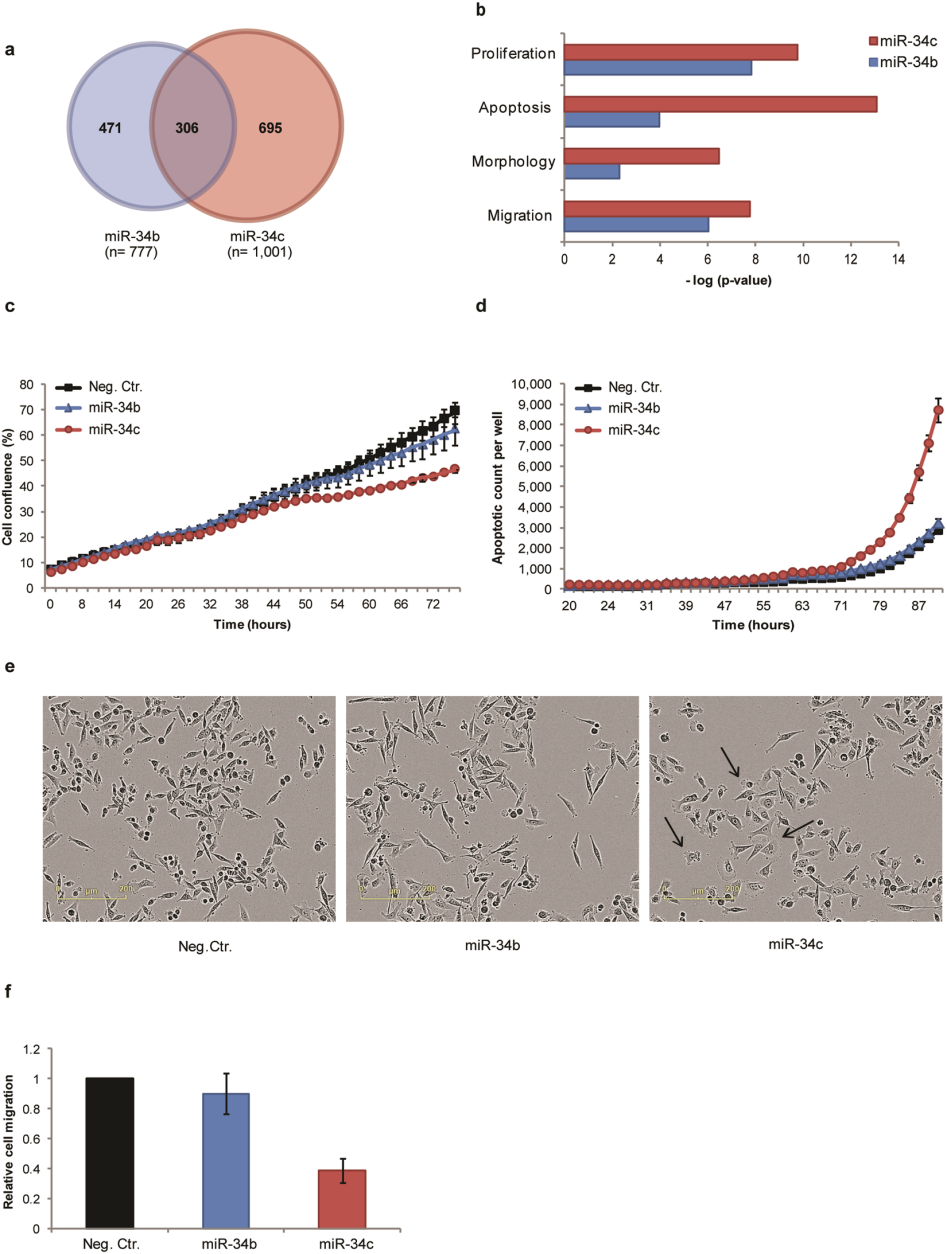
Comparing effects of introducing miR-34b-5p and miR-34c-5p mimics into MDA-MB-231 cells. Cells were transiently transfected with mimics representing miR-34b or miR-34c or a negative control mimic. (**a**) Venn diagram showing the number of significant differentially expressed transcripts (q ≤ 0.05) 48 h after transfection with miR-34 mimics. (**b**) Enriched biological processes based on transcript level changes following introduction of miR-34b (blue bars) or miR-34c (red bars), as determined by Ingenuity Pathway Analysis. (**c**) Cellular growth rate is shown as cell density versus time. One representative experiment is shown (n = 7). Error bars represent standard error of mean (SEM) for technical triplets. (**d**) Apoptosis is shown as number of caspase 3/7-positive cells per well versus time. One representative experiment is shown (n = 5). Error bars represent SEM for technical triplets. (**e**) Phase-contrast images of cells 72 hours after introduction of mimics, magnification 10x. The arrow indicates cells with changed morphology in miR-34c transfected cells. (**f**) The migratory capacity of the transfected cells was determined using a transwell pore assay (n = 2). Data is shown relative to control-transfected cells. Error bars represent SEM for biological experiments.

We separately introduced synthetic mimics representing human annotated versions of the miR-34b-5p (miR-34b) and miR-34c-5p (miR-34c) into the breast cancer cell line MDA-MB-231. The mimics were based on the sequences given in miRBase (Fig. 1). We first examined the global transcription response to each miR-34 mimic by mRNA expression profiling using microarray 48 hours after transfection. The analysis revealed that the levels of 777 and 1001 transcripts significantly changed upon introduction of miR-34b and miR-34c, respectively (Supplementary data S1). 305 transcripts were regulated in the same manner by both mimics (e.g. up- or downregulated by both mimics). Only one transcript had opposite changed expression level, THBS1 that were downregulated by miR-34b and upregulated by miR-34c (Fig. 2a). A number of validated miR-34 family mRNA targets, such as cyclin-dependent kinase 4 (*CDK4*)^8^, cyclin-dependent kinase 6 (*CDK6*), lymphoid enhancer-binding factor 1 (*LEF1*)^16^, Met Proto-oncogene (*MET*)^17^ and *NOTCH1* ^18^ were among the 305 transcripts that were downregulated by both mimics. To further validate the gene lists, we compared the transcripts that were changed by miR-34 mimics with genes identified as predicted targets of the miR-34 family using TargetScan software (version 7.1). We found that 17% of the transcripts down-regulated by miR-34c were predicted miR-34a/c-5p family targets, whereas only 0.9% of the up-regulated transcripts were predicted miR-34c targets. In contrast, for miR-34b 40% of the down-regulated transcripts were predicted miR-34b-5p targets and 14% of the up-regulated transcripts were predicted miR-34b-5p targets (Supplementary data S2).

Importantly, we observed that the majority of transcripts with significantly changed levels were specific for each miR-34 isoform. To identify the biological pathways likely to be affected by the observed expression changes, we performed gene enrichment analyses using Ingenuity Pathway Analysis (IPA) for the response to each mimic. The analysis predicted changes in several biological functions known to be regulated by the miR-34 family, such as cell growth, apoptosis, cell morphology and cell migration (Fig. 2b and Supplementary data S3).

Gene expression analyses of miR-34b and miR-34c transfected cells identified a number of genes involved in cellular proliferation (165 and 212 genes, p = 1.49E-08 and p = 1.71E-10, respectively). 76 mRNAs were regulated by both miR-34b and miR-34c, all of these were changed in a similar manner. We therefore investigated the effect of miR-34 mimics on cell growth in MDA-MB-231 cells. We observed a reduced cell growth capacity after transfection with miR-34c, while miR-34b only had a minor, delayed effect on cell growth (Fig. 2c).

We subsequently investigated the effect of the mimics on apoptosis. IPA analysis revealed that miR-34c regulated 167 transcripts implicated in apoptosis, while miR-34b affected 105 transcripts involved in the same process (p = 8.39E-14 and p = 1.10E-04, respectively). 52 transcripts were regulated by both miR-34b and miR-34c, all of these were regulated in a similar manner. Our experiments showed that caspase-3/7 activity was induced in miR-34c-transfected cells approximately 72 hours after transfection, while transfection with the miR-34b mimic did not induce caspase-3/7 activity (Fig. 2d).

The IPA analyses revealed that miR-34c affected the expression levels of 50 genes involved in cell morphology while miR-34b only affected 30 (p = 3.46E-07 and p = 5.09E-03, respectively). 15 transcripts were regulated by both miR-34b and miR-34c, all of these were regulated in a similar manner. In line with this, we observed that miR-34c had a profound effect on the cellular morphology of MDA-MB-231 cells 72 hours after transfection, whereas no clear morphological changes were observed for miR-34b transfected cells (Fig. 2e). More specifically, in contrast to the spindle-shaped, mesenchymal-like morphology of untreated and control treated cells, the cells transfected with miR-34c appeared rounder, with a more transparent cytoplasm. We observed changed expression of 101 genes known to be involved in cell migration upon introduction of miR-34c (p = 1.78E-08), while only 78 (p = 9.44E-07) genes were affected by over-expression of miR-34b. 35 transcripts were regulated by both miR-34b and miR-34c, all of these were changed in the same direction. In line with this, we found a clear reduction in the migratory capacity following transfection with miR-34c, whereas transfection with miR-34b did not result in any change in migratory capacity compared to the control (Fig. 2f).

### Investigation of miR-34 levels and sequences

**Figure 3:**
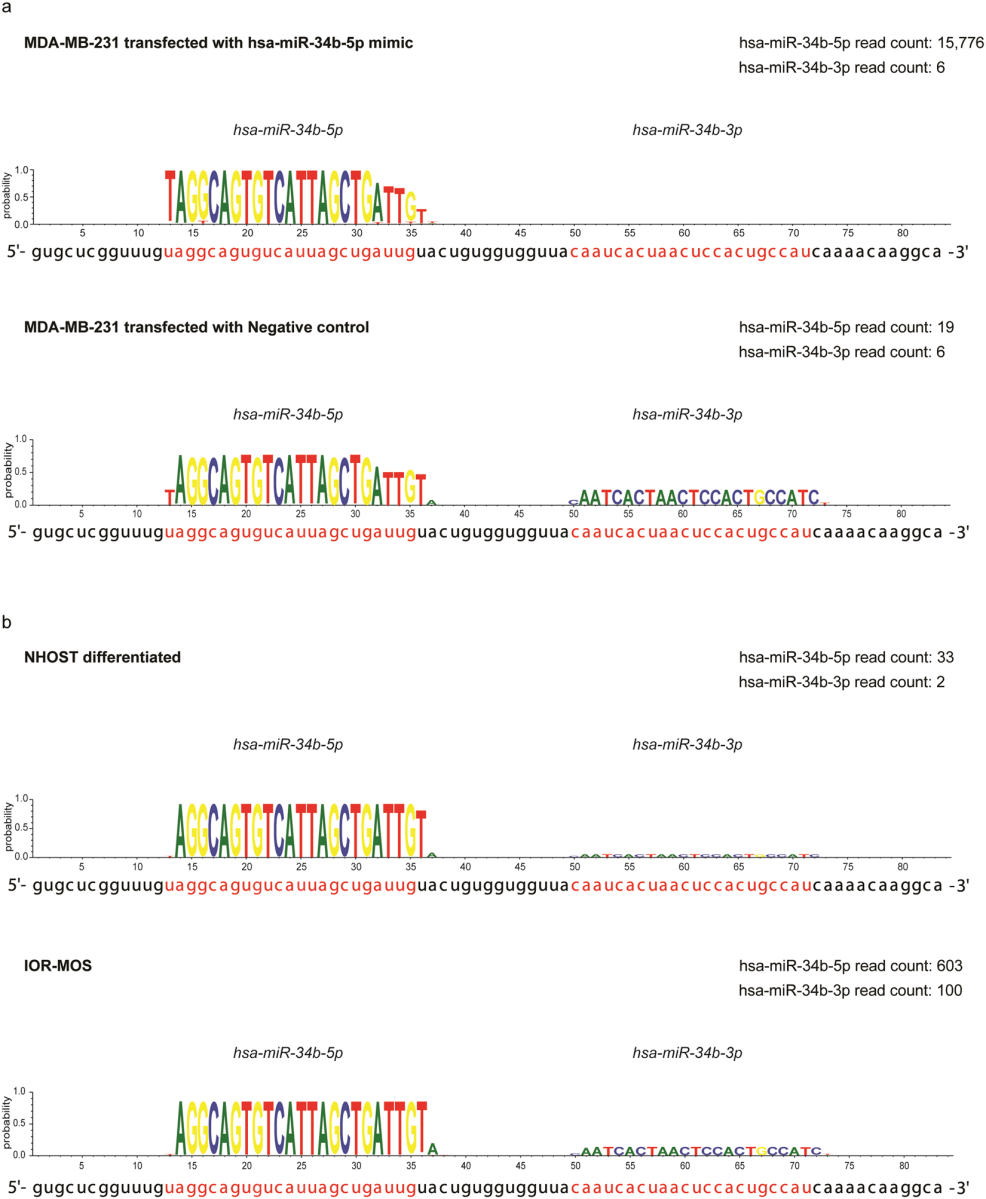
Sequencing data for miR-34b. (**a**) The mature miR-34b sequences obtained from MDA-MB-213 cells 48 hours post transfection with miR-34b mimics (upper panel) or control mimics (lower panel). (**b**) The mature miR-34b sequences obtained from the noncancerous mesenchymal stromal cells (NHOST) after osteogenic differentiation and the osteosarcoma cell line IOR-MOS. All reads were mapped using miRDeep2 to identify mature miRNA sequences, and visualized by the WebLogo program. The plots show the probability for a given nucleotide at each individual position in the mature miRNA based on the sequencing reads, with the annotated sequences given in red below the plot.

To investigate the endogenous expression of miR-34b and miR-34c in MDA-MB-231 cells and to evaluate the transfection efficiency we sequenced small RNAs from control and miR-34b transfected cells. In miR-34b transfected cells, we found an 830-fold increase in miR-34b-5p reads compared to control-transfected cells, indicating that the transfection was highly efficient. Reads with heterogeneous 3′ termini were observed for both endogenous miR-34b-5p and the transfected mimics. In cells transfected with miR-34b we found three major groups of miR-34b-5p sequences. Of the total reads, 22.4% were identical to the synthetic mimic, 17.9% had an additional uridine (U) at the 3′ terminus and 19.4% lacked four nucleotides at the 3′ terminus (Fig. 3a and Supplementary data S4). The remaining 40% of the reads lacked one to three nucleotides in 3′ ends and/or contained single base substitutions.

We found that miR-34c was expressed at a higher level than miR-34b in control-transfected MDA-MB-231 cells (342 versus 19 reads, respectively, Supplementary data S5). Both the 5p and the 3p miRNA arms were detected for endogenous miR-34b and miR-34c, with the 5p arm as the major product, accounting for 76% and 99.7%, respectively (Supplementary data S5). The annotated miR-34c-5p was in accordance with the observed miR-34c sequences, both at the 5′ and the 3′ end (95%). Surprisingly, we found that endogenous miR-34b-5p in MDA-MB-231 cells did not match the annotated miR-34b-5p sequence. The endogenous miR-34b-5p lacked the first annotated 5′ nucleotide and had an additional nucleotide at the 3′ end (Fig. 3a and Supplementary data S5). Thus the expressed mature miR-34b-5p (hereafter referred to as miR-34b-5p’) was still 23 bases long, but the seed sequence was shifted one position (spanning nucleotides 2–8). Interestingly, the miR-34b-5p’ seed sequence was identical to those annotated for miR-34a and miR-34c. We subsequently investigated whether the miR-34b-5p’ also was expressed in other cell types than MDA-MB-231 cells. This was done by analysing our sequence data from other cancer cell lines (one liposarcoma and four osteosarcoma cell lines), a non-cancerous mesenchymal stromal cell line and an osteoblast primary cell culture (Fig. 3b and Supplementary data S6 and S7). Interestingly, more than 95% of miR-34b-5p reads in the samples analysed matched the miR-34b-5p’ sequence found in MDA-MB-231 cells, compared to less than 2% matching the annotation. In addition, we evaluated human miR-34b-5p sequence data from miRBase, which contains data from embryonic stem cells, uterine cervix and melanoma^19–21^. Here 97 RPM (reads per million) matched the miR-34b-5p’ (equivalent to 50%), and only 3 RPM (equivalent to 1.5%) matched the annotated miR-34b-5p form. Moreover, 30% of the reads were not full length at the 3′ end, but also for these sequences the large majority lacked the annotated 5′ U. 10% of the reads were not full length at the 5′ end, thus lacking part of the seed sequence.

### MiR-34b-5p’ function closely resembles that of miR-34c

**Figure 4:**
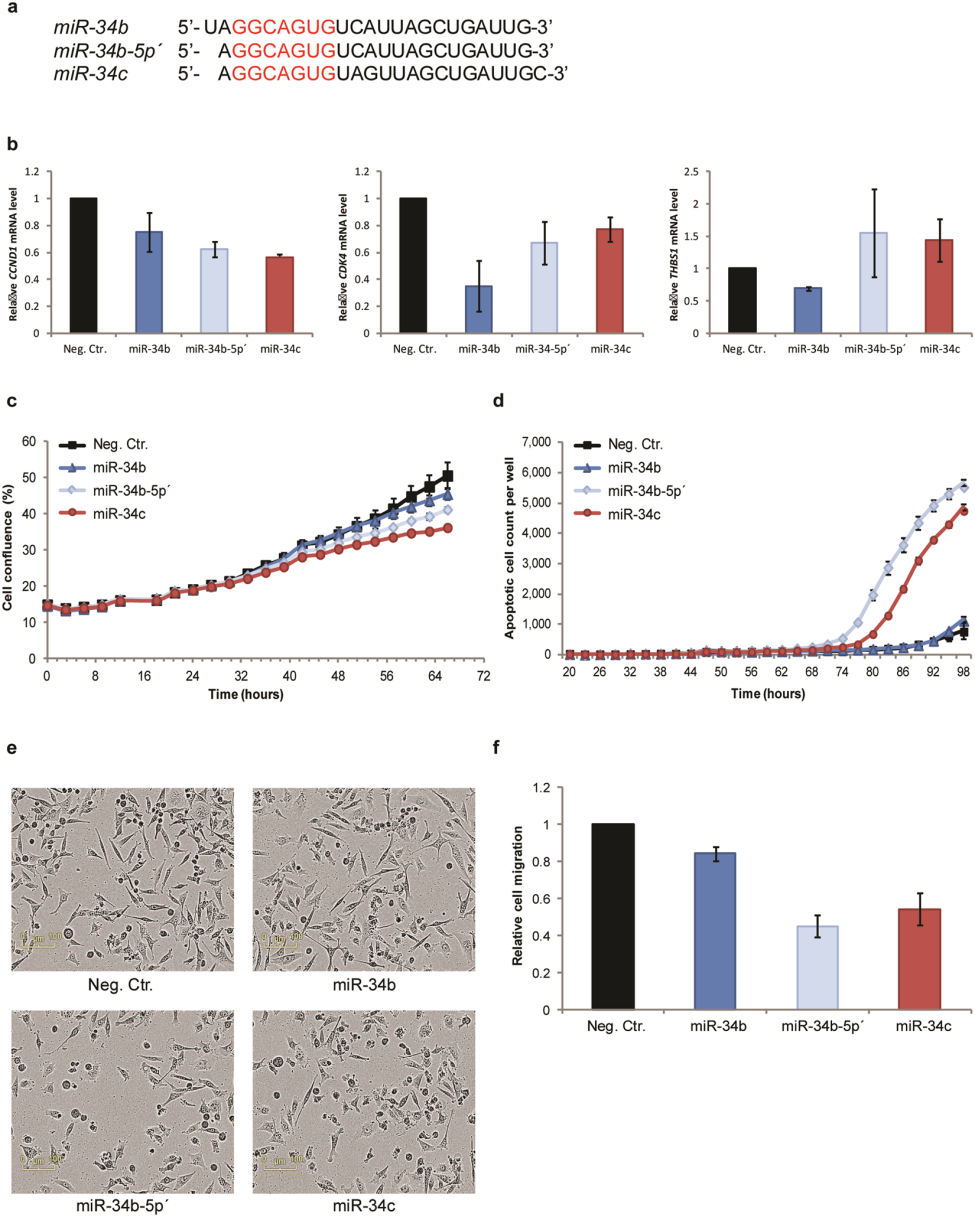
Comparing effects of the annotated and expressed miR-34b isoform. (**a**) Alignment of the three synthetic miR-34 isoforms with the miR-34 family seed sequence highlighted in red. (**b**) Relative mRNA expression levels of CCND1, CDK4 and THBS1 48 hours after introduction of miR-34b, miR-34b-5p’or miR-34c mimics (n = 2). Error bars represent SD for biological experiments (**c**) Cellular growth rate is displayed as cell density versus time. One representative experiment is shown (n = 3). Error bars represent standard error of mean (SEM) of values for technical triplets. (**d**) Apoptotic cells were determined based on caspase3/7 activity over time. One representative experiment is shown (n = 3). Error bars represent SEM for technical triplets. (**e**) Phase-contrast images of cells 72 hours after introduction of mimics, magnification 10x. (**f**) The migratory capacity of the cells 48 h post transfection was evaluated in a transwell migration assay (n = 3). Error bars represent SEM for biological experiments.

To investigate the functions of miR-34b-5p’ and compare it to that of miR-34b and miR-34c, we individually transfected MDA-MB-231 cells with each of the three isoforms (mimic sequences shown in Fig. 4a). We first assessed the expression of three selected miR-34 targets by qPCR. *CDK4* and cyclin D1 (*CCND1*) were chosen since these are validated miR-34 targets^8, 22^. Thrombospondin 1 (*THBS1*) expression was analysed since this transcript was predicted to be a target of the miR-34b-5p both by the TargetScan and by the MiRDB databases, and furthermore, the microarray data showed that *THBS1* expression was reduced in miR-34b transfected cells, but up-regulated in cells transfected with miR-34c. Overexpression of any of the miR-34 isoforms decreased the transcript levels *CDK4* and *CCND1* 48 h after transfection (Fig. 4b). Interestingly, in contrast to the miR-34b mimic, which as predicted reduced the mRNA levels of *THBS1*, an increase of mRNA levels was observed following introduction of either miR-34b-5p’ or miR-34c mimic. Furthermore, miR34b-5p’ reduced the growth of MDA-MB-231 cells more efficiently than miR-34b, although not quite as strong as observed for miR-34c (Fig. 4c). Moreover, miR-34b-5p’, unlike the mimic representing the annotated miR-34b, induced apoptosis as indicated by increased caspase 3/7 activity, at even higher level than observed for cells transfected with miR-34c (Fig. 4d). In addition, unlike cells transfected with miR-34b, cells transfected with miR-34b-5p’ changed morphology, closely resembling cells transfected with miR-34c (Fig. 4e). We also performed transwell cell migration assay to evaluate the effect of ectopically expressed miR-34b-5p’ and found that cellular migration was reduced after treatments with all three isoforms, more efficiently for both miR-34b-5p’ and miR-34c (Fig. 4f).

## Discussion

In our study, we found that mimics representing the annotated versions of human miR-34b-5p (miR-34b) and miR-34c-5p (miR-34c) affected different gene sets, and importantly, resulted in different cellular effects. MiR-34c had the strongest anti-tumorigenic effect in all the evaluated biological functions, reducing cell growth and cell migration as well as inducing apoptosis. In contrast, the annotated miR-34b had a weak or no effect on these functions. These findings were in conflict with published data comparing miR-34 family members^8^ and led us to sequence the endogenous miR-34b. To our surprise, the annotated miR-34b was hardly expressed in any of the investigated cells. Instead, miR-34b-5p’ was the dominant variant in both cancerous (breast, sarcoma, melanoma, gastric and cervix cancer) and non-cancerous cells (mesenchymal stromal cells, osteoblasts, embryonic stem cells, gastric cells and normal cervix)^19–21, 23^. We have demonstrated that miR-34b-5p’ displays the same tumour suppressive activity as miR-34c, by functional assays evaluating cell growth, induction of apoptosis and cell migration, which is in accordance with the results from He *et al*.^8^. Surprisingly, the miR-34b annotation appears to be based on very limited data. In fact, the initial cloning and sequencing of miR-34b was performed using mouse embryonic stem cells. Three libraries were sequenced, and miR-34b was identified as a single clone in only one of the libraries^24^. For several species the original miR-34b-5p annotation is corrected, including mouse and gorilla, and is there in accordance with the miR-34b-5p’ identified here. Notably, the miR-34b-5p’ sequence is the annotated isoform of human miR-34b in a novel database (MiRGeneDB) in which miRNA genes are manually curated based on deep sequencing data^25^. The miR-449 family (miR-449a, miR-449b, and miR-449c) has the same seed sequence as the miR-34 family, and should therefore be considered part of the miR-34 family although the miR-449 family is currently annotated separately. Interestingly, miRBase also annotates a seed-shifted version for one miR-449 family member (miR-449c), but also here less than 5% of the deep sequencing reads deposited in the miRBase match the annotated miR-449c. A recent study found that the annotated miR-34b and miR-449c represents minor variants in human airway epithelial cell, and propose that the annotated versions represent isomiRs^26^. Altogether, this suggests that the miR-34b-5p’should be the reference miRNA, while the seed shifted version is an isomiR.

Whereas most conserved miRNAs undergo a very precise 5′ end processing, there are reports in the literature describing heterogeneity of the 5′ terminus of mature miRNAs^7^. For instance, miR-133a has two equally dominant isomiRs from the same strand, with the difference between the two being a one-nucleotide shift at the 5′ end. The existence of two or more alternative 5′ termini would broaden the regulatory impact of the miRNA. We also noted that both endogenous miR-34b and the transfected mimics had reads with heterogeneous 3′ termini. In particular, many reads had an additional U at the 3′ end. In general, 3′ modifications are observed for the majority of miRNAs in small RNA sequencing data. They can be a result of variation in miRNA processing by Drosha and Dicer or non-template addition of nucleotides^7, 27^. The differences are not thought to affect target specificity of the miRNAs, but may regulate miRNA stability and lead to their degradation. Uridylation, as we found for miR-34b, is known to promote miRNA degradation^28^. Degradation would be consistent with the observation that the levels of miR-34b in most of the samples are lower than miR-34c, even though they originate from the same transcript.

The difference between the isoforms in the position of the seed sequence would be expected to affect target recognition. Recently, the crystallographic structure of the miRNA-mRNA-Argonaut complex revealed that the nucleotides 2–6 of the miRNA are exposed and available for pairing to target mRNA^29^. Consequently, the annotated miR-34b isoform when presenting nucleotide 2–6 will have a different sequence (AGGCA) available for mRNA pairing, compared to miR-34a, miR-34b-5p’ and miR-34c (GGCAG). Interestingly, the exposed nucleotides for miR-34b pairs perfectly to the predicted miR-34 binding site in the 3′ UTR of *THBS1* mRNA, while as a result of the shift in the exposed nucleotides for miR-34b-5p’, this seed sequence is no longer complementary to the target mRNA. In line with this, we found that only the annotated miR-34b isoform reduced the *THBS1* expression. Nevertheless, similarities in the transcriptome changes and repression of *CDK4* and *CCND1* expression are observed, indicating that the annotated miR-34b still recognises some miR-34 target genes. Emerging evidence is indicating that not all RISC-mRNA interactions require a complete seed sequence complementarity^30, 31^. In addition, about 25% of the predicted miR-34b target genes should by chance have the complementary base to the shift in miR-34b 5′ terminus.

The endogenous expression of miR-34a/b/c and miR-449a/b/c is low in MDA-MB-231 cells, with RPM values below 300 for both 5p and 3p strands for all these miRNAs based on sequencing data from control mimic transfected MDA-MB-231 cells (Fig. 3a, Supplementary data S5, and data not shown). These levels are thought to be below the functional level^32^, so it was expected that MDA-MB-231 cells could be targeted by miR-34 replacement therapy. Although miR-34b-5p significantly changed 777 transcripts, including a large number of transcripts involved in proliferation, apoptosis and cell migration, this mimic had hardly any effect on these phenotypes. This clearly shows that some effect on global transcript level can be expected using high level of mimics, but such findings should be interpreted with care, as these might still not affect the phenotypes indicated.

Next generation sequencing has greatly improved our ability to identify the actual mature miRNA sequences, compared to older technologies such as microarrays or qPCR. The currently annotated form of human miR-124 has been subject to a one-nucleotide shift, compared to the initially identified sequence, based on extensive sequencing data^7^. It is likely that many more miRNAs would need to have their annotation adjusted following the large increase in sequencing over the last years, as already suggested by Morin D *et al*.^6^ and more recently by McCall *et al*.^33^. Notably, a number of well-known commercial companies sell miRNA mimics based on the miRBase annotation, thus, such errors might be misleading for validation of target genes and for gain-of-function studies^34–41^.

In conclusion, the miR-34 family is an important group of miRNAs with anti-tumorigenic effects, and the precise knowledge of the expressed isomiRs and their function is critical. As we have shown here, public databases contain misleading or erroneous data, and it is therefore imperative that studies designed to investigate miRNA function first establish the mature sequence of the endogen miRNA of interest. Our findings further confirm that small differences in miRNA sequences, like in the case of seed-shifted isomiRs, may have profound effects on the functional effects of miRNAs.

## Materials and Methods

### Cell lines and culturing

The MDA-MB-231 triple-negative breast cancer cell line was obtained from the American Type Culture Collection (ATCC). The cells were cultured in RPMI-1640 medium (Sigma-Aldrich, Missouri, USA) supplemented with 5% fetal bovine serum (FBS) (Sigma-Aldrich) and 2mM L-glutamate (GlutaMAX, Sigma-Aldrich), referred to a as growth media, at 37 °C in a humidified incubator with 5% CO_2_. The cell line was routinely tested for mycoplasma, and always found negative. The cell line was also subject to short tandem repeat (STR)-DNA profiling of 15 loci and amelogenin (Genotyping core facility, Oslo University Hospital) and matched the STR-profile available from ATTC. The origin and culturing of mesenchymal stromal cells (NHOST) and the differentiation protocol are described in Håkelien *et al*.^42^. The origin and culturing of osteoblast primary culture (OB4), liposarcoma (SA-4) and osteosarcoma cell lines (MHM, IOR-OS18, SAOS2, IOR-MOS) are described in Lorenz *et al*.^43^.

### Transient transfection of miRNA mimic

MDA-MB-231 cells were plated at 3 × 10^3^ cells per well in 96-well plates or 7 × 10^5^ cells per well in 12-well plates the day before transfection. The synthetic pre-miRNA precursors (Ambion, Grand Island, USA) hsa-miR-34b-5p (PM10743), hsa-miR-34c-5p (PM 11039), hsa-miR-34b-5p’ (AM17103) and Negative Control #2 oligos (AM 17111) were transiently transfected into the cells at a final concentration of 18 nM using the lipidic transfection agent INTERFERin (PolyPLUS, Illkirch, France), according to the manufacturer’s protocol. One day after transfection, the growth medium containing the transfection agent was replaced with fresh growth medium. The mimics are small, partially double-stranded RNAs. These are generated by annealing two single-stranded RNA oligonucleotides, where one strand is identical to the mature miRNA of interest. This is the strand that will be incorporated into the RISC, and is the sequences given in Fig. 4a.

### Cell growth

Cell confluence was monitored using the IncuCyteZoom Kinetic Imaging System (Essen BioScience, Birmingham, UK), a live-cell microscopy system that estimates cell growth over time. Cells were imaged at 10 × magnification at 37 °C, 5% CO_2_ every three hours, and cell confluence (%) was determined based on the phase-contrast pictures obtained. The data is presented as % of cell confluence ± standard error of the mean (SEM). Each condition was performed in technical triplicates, and the experiment repeated at least three times.

### Apoptosis analysis

CellPlayer 96-Well Kinetic Caspase-3/7 reagent (Essen Bioscience) (1:3000 dilution) was included in the fresh media added to the cells one day post transfection, to quantitative measure caspase 3/7 activity by fluorescent live cell imaging (488 nm) over time in the IncuCyteZoom (Essen Bioscience). Data was analysed using IncuCyteZoom analysis software (Essen BioScience) to detect and quantify number of apoptotic cells per well, as well as total number of cells represented by cell confluence (%). Each condition was performed in technical triplicates, and the experiment was repeated at least three times.

### Transwell migration assay

48 hours post transfection cells were harvested by trypsinisation and washed once with PBS. 1.8 × 10^4^ cells in serum-reduced media (RPMI-1640 supplemented with 1% FBS and 2 mM GlutaMAX) were seeded per cell culture inserts (8 µm pore size, Falcon, Life Science, USA) placed in the wells of 24-well culture plates. To the lower chamber, RPMI-1640 medium supplemented with 10% FBS and 2 mM glutamine was added as chemo-attractant. After 24 hours incubation at 37 °C with 5% CO_2_, non-migrating cells were removed by “swabbing” the upper part of the membrane and the migratory cells on the lower part of the membrane were fixed, stained with eosin and azure (Hemacolor rapid staining smear kit, Merck Millipore,) and photographed with the inverted microscope Olympus IX81 (Olympus Corporation, Tokyo, Japan) using a 10x objective. Nine pictures, evenly distributed across the insert, were manually counted and an average number of migratory cells per insert were calculated. Only cells with visible nuclei, including cells along two of the borders with <50% visible nuclei, were included in the count. Each condition was performed in doublets. Results are presented as mean fold-change relative to negative control ± standard error of mean (SEM) of three independent experiments.

### mRNA expression profile and analysis

Total RNA was isolated from three independent biological experiments 48 hours after transfection using the TriReagent (Sigma-Aldrich) according to manufacturer’s protocol. The RNA purity and quantity was measured on a NanoDrop ND-1000 spectrophotometer (Nanodrop Technologies, Delaware, USA), and RNA integrity was evaluated using an Agilent 2100 Bioanalyzer (Agilent Technologies Inc., California, USA). 500 ng of total RNA was used to make biotin-labelled and amplified cRNA with the Illumina TotalPrep-96 RNA Amplification kit (Life Technologies). cRNA was hybridized to Illumina’s Human HT 12 v4 Expression BeadChip (Illumina Inc., California, USA), according to manufacturer’s protocol by the Oslo University Hospital Genomics Core Facility (oslo.genomics.no). Expression values were annotated using the HumanHT-12_V4_0_R2_15002873_B.bgx (Illumina). The expression data was quantile normalized^44^ and log2-transformed, before a rank product^45^ analysis was performed in J-Express (Molmine) using a q value < 0.05 as cut-off to identify significant changes in gene expression. Functional enrichment analysis of differentially expressed genes was performed in Ingenuity Pathway Analysis (IPA) (Ingenuity Systems, USA). Differentially expressed transcripts were uploaded into IPA, and analysed for enrichment in pathways, canonical pathways, biological functions and upstream regulators using default settings.

### miRNA sequencing

Small RNA sequencing libraries were prepared using the IlluminaTruSeq Small RNA Sample Preparation protocol at the Oslo University Hospital Genomics Core Facility. One µg of total RNA was ligated to 3′- and 5′- RNA adapters, and reverse transcribed to generate cDNA libraries for each sample. Libraries were PCR amplified, pooled and size selected before quantified by real-time PCR. Small RNA libraries were sequenced as single read 50 bp using an Illumina HiSeq. 2500 (Illumina) instrument. Real-time analysis, base calling and filtering of low quality reads were done by Illumina’s software packages (SCS2.9/RTA1.9 and Off-line Basecaller v1.9). Fastq files for each sample were analysed using the software package miRDeep2 to map the sequencing reads to the human genome (hg19), identify miRNAs (miRBase, http://www.mirbase.org/) and normalize the expression values (read count normalization) for expression profiling across samples. The miRDeep data were presented using the web-based application WebLogo^46^.

### Quantitative Polymerase Chain Reaction (qPCR)

48 hours post transfection, total RNA was isolated and cDNA synthesis was prepared with the Cell-to-Ct kit for mRNA (Ambion), according to manufacturer’s protocol. For gene expression analysis of *CDK4* (assay ID: Hs00364847_m1), *CCND1* (assay ID: Hs00765553_m1) and *THBS1* (assay ID: Hs00962914_m1), qPCR was performed using TaqMan Gene Expression Assay (Applied Biosystems, California, USA) on the 7900 HT Fast Real-time PCR System (Applied Biosystems). Relative expression was analysed using the comparative threshold cycle (2^−ΔΔCt^) method using TATA-box binding protein (*TBP*) (assay ID: 4326322E) as the endogenous reference gene and the negative control sample as an endogenous control sample for normalization. Normalized gene expression is presented relative to negative control transfected cells ± SD of three independent experiments.

### Data availability

The microarray datasets generated during the current study are available in the GEO repository, with accession number GSE98601. The miR-34b sequencing data analyzed during this study are included in this published article (and its Supplementary Information files).

## Electronic supplementary material

Supplementary data S1

Supplementary data S2

Supplementary data S3

Supplementary data S4-S7
