# Supplementary material for: Analysis of the miR-34 family functions in breast cancer reveals annotation error of miR-34b
Source: Sci Rep. 2017 Aug 28;7:9655. doi: 10.1038/s41598-017-10189-1 (PMC5573726; doi:10.1038/s41598-017-10189-1)
Supplement: Supplementary file 4 — Supplementary data S4-S7 [file 41598_2017_10189_MOESM4_ESM.pdf]

Analysis of the miR-34 family functions in breast cancer reveals annotation error of miR-34b

Authors:
Engkvist ME[1], Stratford EW[1], Lorenz S[1,2], Meza-Zepeda LA[1,2], Myklebost O[1,3]*, Munthe E[1]*
*shared last authorship

[1]Department of Tumour Biology, Institute for Cancer Research, Oslo University Hospital

[2]Department of Core Facilities, Institute for Cancer Research, Oslo University Hospital

[3]Department of Clinical Science, University of Bergen

Cell line : MDA-MB-231 cells transfected with hsa-miR-34b-5p mimic
miRBase precursor : hsa-mir-34b
Total read count : 15782
hsa-miR-34b-3p read count : 6
hsa-miR-34b-5p read count : 15776
remaining reads : 0

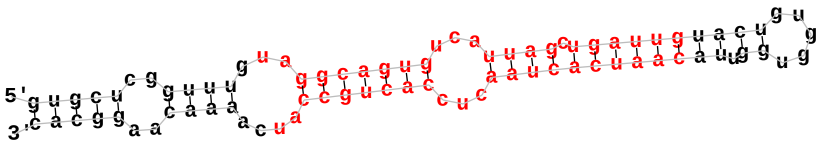

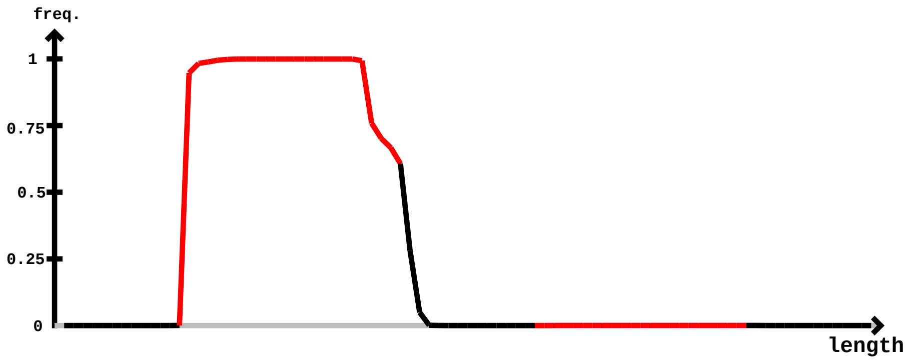

hsa-miR-34b-5p

hsa-miR-34b-3p

5'- gugcucgguuuguaggcagugucauuagcugauuguacugugguggguuacaaucacuaacuccacugccaucaaaacaaggcac -3'	exp		
(((((..((((...(((((((...((((.((((((((((.....)).))))))))))))...)))))))....))))..)))))	reads	mm	sample
..........uCuaggcagugucauuagcugauugu...	1	1	seq
...........Cuaggcagugucauuagcug..	1	1	seq
...........Uuaggcagugucauuagcug..	1	1	seq
...........Uuaggcagugucauuagcugauu...	2	1	seq
...........Auaggcagugucauuagcugauug..	1	1	seq
...........Uuaggcagugucauuagcugauug..	1	1	seq
...........Uuaggcagugucauuagcugauugu...	1	1	seq
............uaggcagugucauAagcu...	1	1	seq
............uaggcagugucauGagcu...	2	1	seq
............uaggcagugucauuagcA...	1	1	seq
............uaggcagGgucauuagcu...	1	1	seq
............uaggcagugCcauuagcu...	1	1	seq
............uagUcagugucauuagcu...	6	1	seq
............Aaggcagugucauuagcu...	1	1	seq
............uaggcagugucauuagcu...	90	0	seq
............uaggcagugucaAuagcug..	3	1	seq
............uaggcagugucUuuagcug..	9	1	seq
............uaggcagugucauuUgcug..	6	1	seq
............uaggcagugGcauuagcug..	3	1	seq
............uUggcagugucauuagcug..	11	1	seq
............uaggcaguUucauuagcug..	5	1	seq
............uagUcagugucauuagcug..	236	1	seq
............Aaggcagugucauuagcug..	6	1	seq
............uaggcagugucauuagcuU..	18	1	seq
............uaggcagugucauuagUug..	3	1	seq
............uaggcagugucauuagcug..	3073	0	seq
............uaggcagugAcauuagcug..	2	1	seq
............uaggcagugucauuaUcug..	7	1	seq
............uaggcaUugucauuagcug..	5	1	seq
............uaUgcagugucauuagcug..	13	1	seq
............uaggcagugucauAagcug..	12	1	seq
............uaggcagugucauuagcGg..	1	1	seq
............uaggUagugucauuagcug..	7	1	seq
............uaggcagAgucauuagcug..	1	1	seq

```
            hsa-miR-34b-5p
                                                 hsa-miR-34b-3p
gugcucgguuuguaggcagugucauuagcugauuguacugugguggguuacaaucacuaacuccacugccaucaaaacaaggcac

............uagCcagugucauuagcug.....................................................    6          1          seq
............uaggcUgugucauuagcug.....................................................    4          1          seq
............uaggcCgugucauuagcug.....................................................    4          1          seq
............uaggcagGgucauuagcug.....................................................    4          1          seq
............Caggcagugucauuagcug.....................................................    1          1          seq
............uaggcagugCcauuagcug.....................................................    1          1          seq
............uaggcaguguUauuagcug.....................................................    3          1          seq
............uaggcagugucauuagcAg.....................................................    1          1          seq
............uaggcagugucauGagcug.....................................................    4          1          seq
............uaggcagugucauCagcug.....................................................    3          1          seq
............uaggcagugucauuagcuA.....................................................    4          1          seq
............uaggcagugucauuaAcug.....................................................    1          1          seq
............uaggcagugucaCuagcug.....................................................    2          1          seq
............uaggcagugucaGuagcug.....................................................    4          1          seq
............uaggcagCgucauuagcug.....................................................    1          1          seq
............Gaggcagugucauuagcug.....................................................    8          1          seq
............uaggcagugGcauuagcuga....................................................    1          1          seq
............uaggcagugucauCagcuga....................................................    2          1          seq
............uaggUagugucauuagcuga....................................................    3          1          seq
............uaggcagugucauuagcCga....................................................    1          1          seq
............uaggcagugucUuuagcuga....................................................    3          1          seq
............uaggcagugucaAuagcuga....................................................    1          1          seq
............uaggcagugucaGuagcuga....................................................    1          1          seq
............uaggcaUugucauuagcuga....................................................    1          1          seq
............uaggcagugucauuagcuga....................................................    679        0          seq
............uaggcagugucauuagcugU....................................................    91         1          seq
............uagCcagugucauuagcuga....................................................    2          1          seq
............Aaggcagugucauuagcuga....................................................    3          1          seq
............uaggcCgugucauuagcuga....................................................    1          1          seq
............uaggcagugucauAagcuga....................................................    3          1          seq
............uagUcagugucauuagcuga....................................................    52         1          seq
............uaggcagugucauuagUuga....................................................    2          1          seq
............uaggcagugucauuagcuUa....................................................    7          1          seq
............uUggcagugucauuagcuga....................................................    2          1          seq
............uaggcagugucauuGgcuga....................................................    2          1          seq
............uaggcagugucaCuagcuga....................................................    2          1          seq
............uaggcaguUucauuagcuga....................................................    1          1          seq
............uaggcagugucauuaUcuga....................................................    2          1          seq
............uaUgcagugucauuagcuga....................................................    5          1          seq
............uaggcagugucauuagcuUau...................................................    2          1          seq
............uaggcagugucauuagcuAau...................................................    1          1          seq
............uaggcagugucauuagcugaC...................................................    18         1          seq
............uaggcagugucauuagcugGu...................................................    1          1          seq
............uaggcagugucauuagcugaA...................................................    2          1          seq
............uagUcagugucauuagcugau...................................................    6          1          seq
............uaggcagugucauuagcugUu...................................................    456        1          seq
............uaggcagugucauuagcugau...................................................    55         0          seq
............uaggcagugucauGagcugauu..................................................    1          1          seq
............uaggcUgugucauuagcugauu..................................................    1          1          seq
............uaggcagugucauuagcugauu..................................................    647        0          seq
............uaggcagugucauuagcugaAu..................................................    1          1          seq
............uaggcagugucUuuagcugauu..................................................    2          1          seq
............uaggcagugucauuagUugauu..................................................    15         1          seq
............uaggcagugucauuGgcugauu..................................................    2          1          seq
............uaggcagugucauuaUcugauu..................................................    11         1          seq
............uaggcagugucaGuagcugauu..................................................    1          1          seq
............uaUgcagugucauuagcugauu..................................................    6          1          seq
............uaggcagugucauuagcGgauu..................................................    2          1          seq
............uaggcagugucauuagcugUuu..................................................    13         1          seq
............uaggcaguguUauuagcugauu..................................................    1          1          seq
............uUggcagugucauuagcugauu..................................................    3          1          seq
............uagUcagugucauuagcugauu..................................................    58         1          seq
............uaggcagugucauuagcugaCu..................................................    77         1          seq
............uaggcagugucauCagcugauu..................................................    1          1          seq
............uagAcagugucauuagcugauu..................................................    2          1          seq
............uaggcagugucauuagcugauG..................................................    23         1          seq
............uaggcagugucauuagGugauu..................................................    1          1          seq
............uaggcaguAucauuagcugauu..................................................    2          1          seq
............uaggUagugucauuagcugauu..................................................    1          1          seq
```

hsa-miR-34b-5p

hsa-miR-34b-3p

gugcucgguuuguaggcagugucauuagcugauuguacuguggugguuacaaucacuaacuccacugccaucaaaacaaggcac

............uaggcagugucauAagcugauu..	4	1	seq
............uaggGagugucauuagcugauu..	3	1	seq
............uaggcagugucauuagcugauA..	24	1	seq
............uaggcagGgucauuagcugauu..	2	1	seq
............uaggcagugucauuagcuUauu..	3	1	seq
............uagCcagugucauuagcugauu..	1	1	seq
............uaggcaUugucauuagcugauu..	4	1	seq
............uaggcagugucauuagcugauug...	3541	0	seq
............uaggcagugucauuagGugauug...	10	1	seq
............uaggcagugucUuuagcugauug...	12	1	seq
............uaggcagGgucauuagcugauug...	3	1	seq
............uaggcagugucauGagcugauug...	6	1	seq
............uaggcagugucauuagcugauuC...	24	1	seq
............uaggcagugucGuuagcugauug...	1	1	seq
............uaggcagugucauuagcugaAug...	1	1	seq
............uaggcagugucauuaUcugauug...	18	1	seq
............uaggcagugucauuagcugauCg...	3	1	seq
............uaggcagugucauuagcugauuU...	543	1	seq
............uaggcagugucauuagcugauAg...	1	1	seq
............uaggUagugucauuagcugauug...	10	1	seq
............uaggcagCgucauuagcugauug...	1	1	seq
............uaggcagugucauuagAugauug...	1	1	seq
............uaggcagugucauuagcugauuA...	95	1	seq
............uaggcagugucauuUgcugauug...	2	1	seq
............uagCcagugucauuagcugauug...	11	1	seq
............uGggcagugucauuagcugauug...	1	1	seq
............uaggcCgugucauuagcugauug...	3	1	seq
............uaggcagugucauuGgcugauug...	2	1	seq
............uaggcaguAucauuagcugauug...	1	1	seq
............uaUgcagugucauuagcugauug...	17	1	seq
............uaggcaguguUauuagcugauug...	5	1	seq
............uaggcaguUucauuagcugauug...	15	1	seq
............Caggcagugucauuagcugauug...	1	1	seq
............uaggcagugucauuagcuCauug...	1	1	seq
............uaggGagugucauuagcugauug...	2	1	seq
............uaggcUgugucauuagcugauug...	2	1	seq
............uaggcagugGcauuagcugauug...	1	1	seq
............uaggcagugucauuagcugauGg...	4	1	seq
............uaggcagugucaCuagcugauug...	3	1	seq
............uagAcagugucauuagcugauug...	2	1	seq
............uaggcagugucauuagcAgauug...	3	1	seq
............uaggcagugucauAagcugauug...	6	1	seq
............uaggcaUugucauuagcugauug...	5	1	seq
............uaCgcagugucauuagcugauug...	1	1	seq
............uagUcagugucauuagcugauug...	262	1	seq
............uaggcagugucauuagcCgauug...	5	1	seq
............uaggcagugucauuagcugUuug...	17	1	seq
............uUggcagugucauuagcugauug...	9	1	seq
............uaggcagugucCuuagcugauug...	1	1	seq
............uaggcagugucauuagUugauug...	34	1	seq
............Gaggcagugucauuagcugauug...	8	1	seq
............uaggcagAgucauuagcugauug...	3	1	seq
............uaggcagugucauuagcGgauug...	1	1	seq
............uaggcagugucaGuagcugauug...	1	1	seq
............uaggcagugucauuagcugaCug...	1	1	seq
............uaggcagugCcauuagcugauug...	2	1	seq
............uaggcagugucauuagcuUauug...	8	1	seq
............uaggcagugucauCagcugauug...	3	1	seq
............uaggcaguUucauuagcugauugu..	14	1	seq
............uaAgcagugucauuagcugauugu..	1	1	seq
............uaggcaguAucauuagcugauugu..	4	1	seq
............uaggcagugucauuagcGgauugu..	2	1	seq
............Gaggcagugucauuagcugauugu..	9	1	seq
............uaggcagugCcauuagcugauugu..	1	1	seq
............uaggcagGgucauuagcugauugu..	3	1	seq
............uaggcCgugucauuagcugauugu..	1	1	seq
............uaggcagugGcauuagcugauugu..	1	1	seq
............uaggcagugucauuagcugauuUu..	68	1	seq
............uaggcaguguUauuagcugauugu..	7	1	seq

```
                hsa-miR-34b-5p
                                                   hsa-miR-34b-3p
gugcucgguuuguaggcagugucauuagcugauuguacuguggugguuacaaucacuaacuccacugccaucaaaacaaggcac

............uaggcagugucauuGgcugauugu................................................    3     1    seq
............Aaggcagugucauuagcugauugu................................................    2     1    seq
............uaggcagugucauuUgcugauugu................................................    4     1    seq
............uaUgcagugucauuagcugauugu................................................    21    1    seq
............uaggcagugucauuagcugauAgu................................................    2     1    seq
............uaggcagugucauuagcugauuAu................................................    3     1    seq
............uaggUagugucauuagcugauugu................................................    6     1    seq
............uaggcagugucauuagcugaAugu................................................    1     1    seq
............uaggcagugucauuagcugauugA................................................    129   1    seq
............uagAcagugucauuagcugauugu................................................    4     1    seq
............uUggcagugucauuagcugauugu................................................    11    1    seq
............uaggcagugucauuagcugauGgu................................................    1     1    seq
............uagUcagugucauuagcugauugu................................................    273   1    seq
............uaggcagugucauuaUcugauugu................................................    3     1    seq
............uaggcagugucauuagcCgauugu................................................    1     1    seq
............uaggcagugucauuagcuUauugu................................................    5     1    seq
............uaggcagugAcauuagcugauugu................................................    2     1    seq
............uaggcaUugucauuagcugauugu................................................    4     1    seq
............uaggcagugucaGuagcugauugu................................................    1     1    seq
............uaggcagugucauuagcugauuCu................................................    17    1    seq
............uaggcagugucaCuagcugauugu................................................    4     1    seq
............uaggcagAgucauuagcugauugu................................................    4     1    seq
............uaggcagugucauuagcugaCugu................................................    4     1    seq
............uaggcagugucauuagcugUuugu................................................    10    1    seq
............uaggcagugucauuagcugauugC................................................    57    1    seq
............Caggcagugucauuagcugauugu................................................    1     1    seq
............uaggcagCgucauuagcugauugu................................................    1     1    seq
............uaggcUgugucauuagcugauugu................................................    3     1    seq
............uaggcagugucauuagcuAauugu................................................    1     1    seq
............uaggcagugucauuagcugauugu................................................    2832  0    seq
............uaggcagugucauuagcugauugG................................................    6     1    seq
............uaggAagugucauuagcugauugu................................................    1     1    seq
............uaggcagugucauGagcugauugu................................................    1     1    seq
............uaggcagugucGuuagcugauugu................................................    1     1    seq
............uaggcagugucauuagUugauugu................................................    21    1    seq
............uaggcagugucauCagcugauugu................................................    4     1    seq
............uagCcagugucauuagcugauugu................................................    6     1    seq
............uaggcagugucauAagcugauugu................................................    10    1    seq
............uaggcagugucUuuagcugauugu................................................    15    1    seq
............uaggcagugucauuagcugauCgu................................................    1     1    seq
............uaggcagugucauuagcugauuguG...............................................    1     1    seq
............uagAcagugucauuagcugauugua...............................................    1     1    seq
............uagUcagugucauuagcugauugua...............................................    24    1    seq
............uaggcagugucauuagUugauugua...............................................    1     1    seq
............uaggcagugucauuagcugauugGa...............................................    1     1    seq
............uaggcagugucauuagcugauugua...............................................    195   0    seq
............uaggcagugucauuUgcugauugua...............................................    1     1    seq
............uaggcagugucauuagcugauugAa...............................................    4     1    seq
............uaggcagugucauuagcugauuUua...............................................    2     1    seq
............uaggcagugucauuagcuUauugua...............................................    1     1    seq
............uaggcagugucauuagcugauuguC...............................................    27    1    seq
............uaggcaUugucauuagcugauugua...............................................    1     1    seq
............uaggcagugucauuagcugauuguU...............................................    470   1    seq
............uaggcagugucauuaUcugauugua...............................................    1     1    seq
............uaggcagugucauuagcugauuguaA..............................................    3     1    seq
............uaggcagugucauuagcugauugAac..............................................    3     1    seq
............uaggcagugucauuagcugauuguaU..............................................    4     1    seq
............uaggcagugucauuagcugauuguaG..............................................    1     1    seq
............uaggcagugucauuagcugauuguaUu.............................................    2     1    seq
............uaggcagugucauuagcugauugAacu.............................................    1     1    seq
............uaggcagugucauuagcugauuguUcu.............................................    1     1    seq
.............aggcagugucaGuagcug.....................................................    1     1    seq
.............aggcagugucauuagcuC.....................................................    1     1    seq
.............aggcagugucauuagcug.....................................................    215   0    seq
.............Uggcagugucauuagcug.....................................................    2     1    seq
.............aggcagugucauuUgcug.....................................................    1     1    seq
.............aggcagugucUuuagcuga....................................................    1     1    seq
.............aggcagugucauuGgcuga....................................................    1     1    seq
.............aggcagugucauuagcuga....................................................    26    0    seq
```

hsa-miR-34b-5p

hsa-miR-34b-3p

gugcucgguuuguaggcagugucauuagcugauuguacuguggugguuacaaucacuaacuccacugccaucaaaacaaggcac

.............aggcagugucauuagcugau...	2	0	seq
.............aggcagugucauuagcugUu..	10	1	seq
.............aggcUgugucauuagcugauu..	1	1	seq
.............aggGagugucauuagcugauu..	1	1	seq
.............aggcagugucauuagcugaCu..	3	1	seq
.............aggcagugCcauuagcugauu..	1	1	seq
.............aggcagugucauuagcugauu..	12	0	seq
.............aggUagugucauuagcugauug...	2	1	seq
.............agUcagugucauuagcugauug...	2	1	seq
.............aggcagugGcauuagcugauug...	1	1	seq
.............aUgcagugucauuagcugauug...	4	1	seq
.............aggcagugucauuagcugaAug...	1	1	seq
.............Gggcagugucauuagcugauug...	1	1	seq
.............aggGagugucauuagcugauug...	1	1	seq
.............aggcagugucauuagcugauuA...	1	1	seq
.............aggcaguUucauuagcugauug...	1	1	seq
.............aggcagugucauuagcugauuU...	3	1	seq
.............aggcUgugucauuagcugauug...	2	1	seq
.............aggcagAgucauuagcugauug...	1	1	seq
.............aggcagugucauuUgcugauug...	2	1	seq
.............aggcagCgucauuagcugauug...	1	1	seq
.............aggcagugucauuagcugauug...	174	0	seq
.............Uggcagugucauuagcugauug...	1	1	seq
.............aggcagugucauuagcGgauug...	1	1	seq
.............aggcagugucauuagcCgauugu..	1	1	seq
.............aggcagugucauuagcugauugA..	3	1	seq
.............aggcagugucauuagcugauugC..	2	1	seq
.............aggcagugucauuagcugauuCu..	1	1	seq
.............aggcagugucauuagcugUuugu..	1	1	seq
.............agUcagugucauuagcugauugu..	1	1	seq
.............aggcaguguGauuagcugauugu..	1	1	seq
.............aggUagugucauuagcugauugu..	1	1	seq
.............aggcagugucauuagcugauugu..	48	0	seq
.............aggcagugucauuagcugauuguC...	2	1	seq
.............aggcagugucauuagUugauugua...	1	1	seq
.............aggcagugucauuagcugauuguU...	20	1	seq
.............aggcagugucauuagcugauugua...	8	0	seq
.............aggcagugucauuagcugauuguaU..	1	1	seq
..............ggcagugucauuagcuga..	4	0	seq
..............ggcaguAucauuagcuga..	1	1	seq
..............ggcagugucauuagcugUu...	1	1	seq
..............ggcagugucauuagcugaCu..	1	1	seq
..............ggcagugucauuagcugauu..	2	0	seq
..............ggcagugucauuagcugaGug...	1	1	seq
..............ggcagugucauuaUcugauug...	1	1	seq
..............ggcagugucauuagcugauug...	54	0	seq
..............ggcagugucauuagcugauuU...	1	1	seq
..............ggcagugucauAagcugauug...	1	1	seq
..............ggcGgugucauuagcugauug...	1	1	seq
..............ggcagugucauuagcugaCug...	1	1	seq
..............ggcagugucauuagUugauug...	1	1	seq
..............ggcagugucaGuagcugauug...	1	1	seq
..............ggcagugucauuagcugauugu..	10	0	seq
..............ggcagugucauuagcugauugC..	1	1	seq
..............ggcagugucauuagcugauugua...	1	0	seq
..............ggcagugucauuagcugauuguC...	1	1	seq
...............gcagugucauuagcuUauug...	1	1	seq
...............gcagugucauuagUugauug...	3	1	seq
...............gcagugucauuUgcugauug...	1	1	seq
...............gcagugucauuagcugUuug...	1	1	seq
...............gcagugucauuagcugauug...	88	0	seq
...............gcaUugucauuagcugauug...	6	1	seq
...............gcagugucauuaUcugauug...	1	1	seq
...............gcagugucauuagcugauugu..	2	0	seq
...............gcagugucauuagcugauugua...	1	0	seq
................cagugucauuagcugauu..	3	0	seq
................cagugucauuagcugauA..	1	1	seq
................cagugucauuagcugauuU...	1	1	seq
................cagugucauuagcAgauug...	1	1	seq

hsa-miR-34b-5p

hsa-miR-34b-3p

gɑg ucgguuuguaggcagugucauuagcugauuguacugugguggguuacaaucacuaacuccacugccaucaaaacaaggcac

................cagugucauuagcugauug..	38	0	seq
................cagugucauuaUcugauug..	1	1	seq
................cagugucauuagcugauugu...	2	0	seq
.................agugucauuagcugauuU..	1	1	seq
.................agugGcauuagcugauug..	1	1	seq
.................agugucauuagcugauug..	18	0	seq
.................aguUucauuagcugauug..	2	1	seq
.................agugucauuagcugauugA...	1	1	seq
..................gugucauuagcugauugu...	1	0	seq
..................gugucauuaCcugauugu...	1	1	seq
....caaucacuaacuccacugccauc...........	1	0	seq
....aaucacuaacuccacugccauc...........	3	0	seq
....aaucacuaacuccacugccaucU..........	1	1	seq
....aucacuaacuccacugccauc...........	1	0	seq

Supplementary data S4: miRDeep analysis of miR-34b sequencing data for MDA-MB-231 cells transfected with annotated miR-34b mimic.

The plot shows the percentage of reads for each individual base in the mature miRNA. The annotated sequences are given in red, and the identified sequences and their number of reads are listed below.

Cell line : MDA-MB-231 cells transfected with Negative control
miRBase precursor : hsa-mir-34b
Total read count : 25
hsa-miR-34b-3p read count : 6
hsa-miR-34b-5p read count : 19
remaining reads : 0

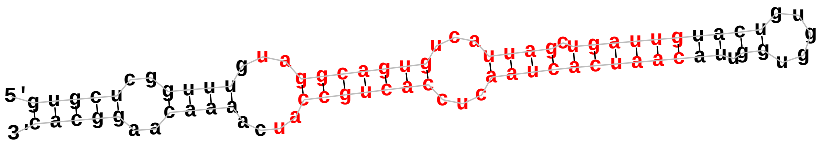

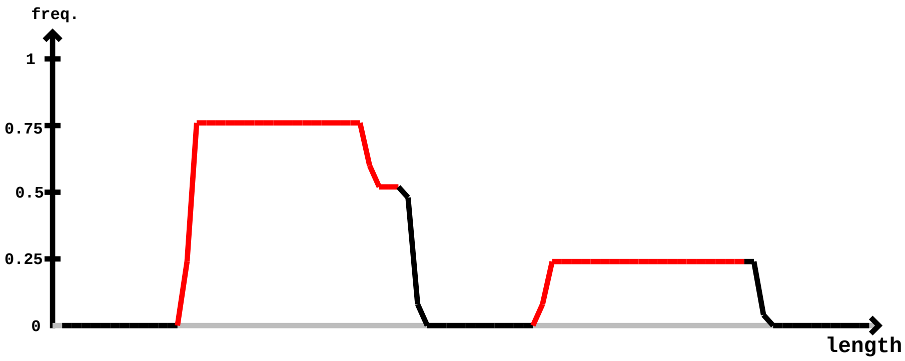

hsa-miR-34b-5p

hsa-miR-34b-3p

```
5'- gugcucgguuuguaggcagugucauuagcugauuguacuguggugguuacaaucacuaacuccacugccaucaaaacaaggcac   -3'    exp
    (((((..((((...(((((((...((((.((((((((((.....)).))))))))))))...)))))))....))))..)))))    reads      mm         sample
    ............uaggcagugucauuagcug......................................................    4          0          seq
    ............uaggcagugucauuagcuga.....................................................    2          0          seq
    .............aggcagugucauuagcugauug..................................................    1          0          seq
    .............aggcagugucauuagcugauugu.................................................    10         0          seq
    .............aggcagugucauuagcugauugua................................................    2          0          seq
    .................................................caaucacuaacuccacugccauc.............    2          0          seq
    ..................................................aaucacuaacuccacugccauc.............    3          0          seq
    ..................................................aaucacuaacuccacugccaucU............    1          1          seq
```

Cell line	: MDA-MB-231 cells transfected with Negative control
miRBase precursor	: hsa-mir-34c
Total read count	: 343
hsa-miR-34c-3p read count	: 1
hsa-miR-34c-5p read count	: 342
remaining reads	: 0

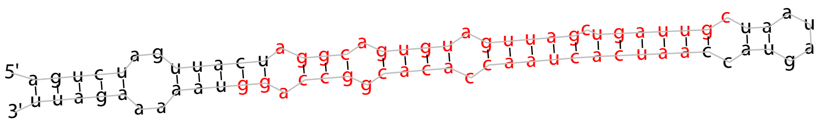

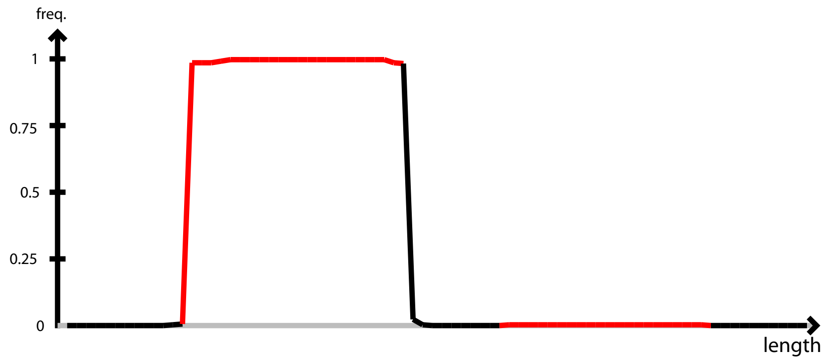

hsa-miR-34c-3p

hsa-miR-34c-5p

5'- agucuaguuacuaggcaguguaguuagcugauugcuaauaguaccaaucacuaaccacacggccagguaaaaagauu -3' exp

.((((..(((((.(((.((((.(((((.((((((.((....)).))))))))))).)))).))).)))))..)))).	reads	mm		sample
..........cuaggcaguguaguuagcugauu...	1		0	seq
...........uaggcaguguaguuagcugauu..	1		0	seq
............aggcaguguaguuagcugauu..	2		0	seq
............aggcaguguaguuagcugauug..	1		0	seq
............aggcaguguaguuUgcugauugc..	1		1	seq
............aggcaguguaguCagcugauugc..	1		1	seq
............aggcaguguaguuagcugauCgc..	1		1	seq
............aggcagugCaguuagcugauugc..	1		1	seq
............aggcagGguaguuagcugauugc..	1		1	seq
............aggcaguguaguuagUugauugc..	1		1	seq
............aUgcaguguaguuagcugauugc..	1		1	seq
............aggcaguguaguuagcugUuugc..	1		1	seq
............aggGaguguaguuagcugauugc..	2		1	seq
............aggcaguguaguuagcugaAugc..	1		1	seq
............aggcagCguaguuagcugauugc..	1		1	seq
............aggcaguguaguuaUcugauugc..	1		1	seq
............aggUaguguaguuagcugauugc..	2		1	seq
............aggcaguguaguuagcugauugc..	305	0		seq
............agUcaguguaguuagcugauugc..	2		1	seq
............aggcUguguaguuagcugauugc..	1		1	seq
............aggcaguguaUuuagcugauugc..	2		1	seq
............aggcaguguaguuagcugauugcC.......................................	3		1	seq
............aggcaguguaguuagcugauugcu.......................................	4		0	seq
............aggcaguguaguuagcugauugcuU......................................	1		1	seq
...............caguguaguuagcugauugc..	1		0	seq
...............Gaguguaguuagcugauugc..	1		1	seq
................aguguaguuagcugauugc..	2		0	seq
....aaucacuaaccacacggccag..........	1		0	seq

Supplementary data S5: miRDeep analysis of miR-34b and miR-34c sequencing data for MDA-MB-231 cells transfected with control mimic.

The plot shows the percentage of reads for each individual base in the mature miRNA. The annotated sequences are given in red, and the identified sequences and their number of reads are listed below.

Cell line : IOR-MOS
miRBase precursor : hsa-mir-34b
Total read count : 703
hsa-miR-34b-3p read count : 100
hsa-miR-34b-5p read count : 603
remaining reads : 0

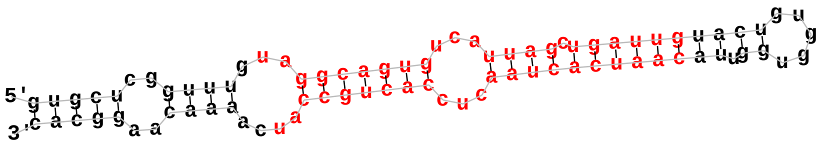

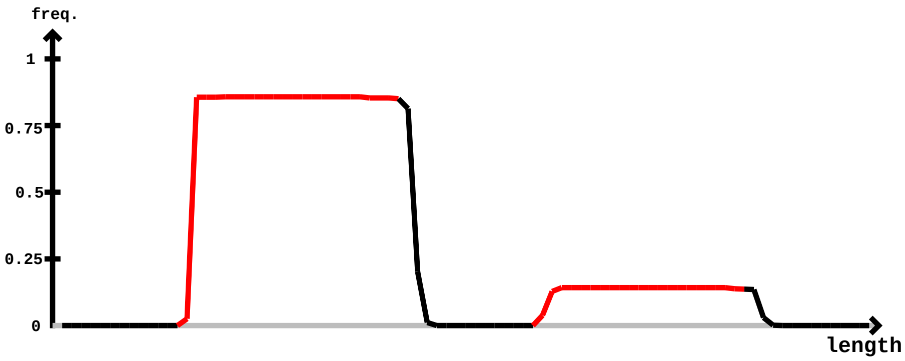

hsa-miR-34b-3p

hsa-miR-34b-5p

```
5'- gugcucgguuuguaggcagugucauuagcugauuguacuguggugguuacaaucacuaacuccacugccaucaaaacaaggcac  -3'   exp
    (((((..((((...(((((((...((((.((((((((((.....)).))))))))))))...)))))))....))))..)))))   reads   mm   sample
    ............uaggcagugucauuagcug.....................................................   2       0    seq
    ............uaggcagugucauuagcugauu..................................................   1       0    seq
    ............uaggcagugucauuagcugauug.................................................   3       0    seq
    ............Caggcagugucauuagcugauug.................................................   1       1    seq
    ............uaggcagugucauuagcugauugu................................................   7       0    seq
    ............uaggcagugucauuagcugauugua...............................................   3       0    seq
    ............uaggcagugucauuagcugauuguaU..............................................   1       1    seq
    .............aggcagugucauuagcug.....................................................   1       0    seq
    .............aggcagugucauuagcugauu..................................................   1       0    seq
    .............aggcagugucauuagcugauug.................................................   21      0    seq
    .............aggcagGgucauuagcugauug.................................................   1       1    seq
    .............aggcagugucauuagcugauugu................................................   415     0    seq
    .............aggcagugucauuagcugauuAu................................................   1       1    seq
    .............aggcagugucauCagcugauugu................................................   1       1    seq
    .............aggcaguguUauuagcugauugu................................................   1       1    seq
    .............agCcagugucauuagcugauugu................................................   2       1    seq
    .............aggcagugucGuuagcugauugu................................................   2       1    seq
    .............aggcagugucauuagcugauuguC...............................................   1       1    seq
    .............aggcagugucauuagcugauuguU...............................................   8       1    seq
    .............aggcagCgucauuagcugauugua...............................................   1       1    seq
    .............aggcagugucauuagcugauugua...............................................   121     0    seq
    .............aggcagugucauuagcugauuguaA..............................................   2       1    seq
    .............aggcagugucauuagcugauuguaU..............................................   5       1    seq
    ................cagugucauuagcugauugu................................................   1       0    seq
    .................................................caaucacuaacuccacugcc...............   2       0    seq
    .................................................caaucacuaacuccacugccU..............   1       1    seq
    .................................................caaucacuaacuccacugccau.............   1       0    seq
    .................................................caaucacuaacuccacugccauc............   18      0    seq
    .................................................caaucacuaacuccacugccauU............   3       1    seq
    .................................................caaucacuaacuccacugccauca...........   1       0    seq
    .................................................caaucacuaacuccacugccaucU...........   1       1    seq
    ..................................................aaucacuaacuccacugcc...............   1       0    seq
    ..................................................aaucacuaacuccacugccauU............   2       1    seq
    ..................................................aaucacuaacuccacugccauc............   46      0    seq
```

```
                                                 hsa-miR-34b-3p
            hsa-miR-34b-5p
gugcucgguuuguaggcagugucauuagcugauuguacuguggugguuacaaucacuaacuccacugccaucaaaacaaggcac

..................................................aaucacuaacuccacugccaucU...........    13    1    seq
..................................................aaucacuaacuccacugccauca...........    1     0    seq
...................................................aucacuaacuccacugccauc............    5     0    seq
...................................................aucacuaacuccacugccauca...........    4     0    seq
...................................................aucacuaacuccacugccaucaa..........    1     0    seq
```

Cell line : NHOST differentiated
miRBase precursor : hsa-mir-34b
Total read count : 35
hsa-miR-34b-3p read count : 2
hsa-miR-34b-5p read count : 33
remaining reads : 0

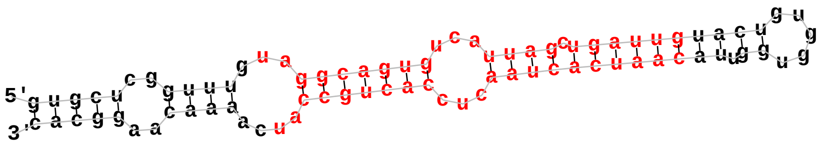

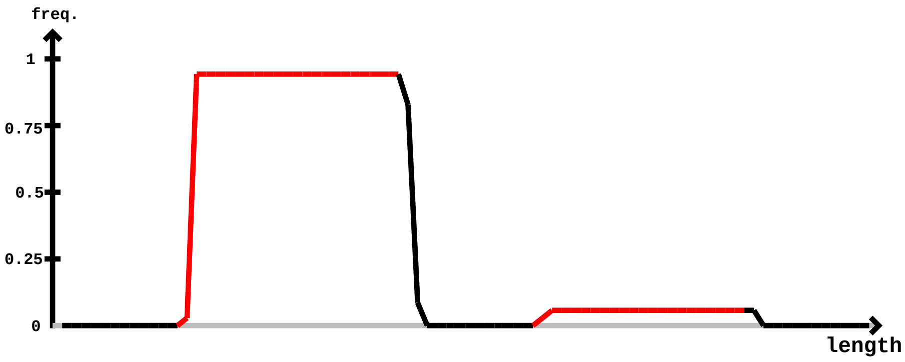


```
                                                  hsa-miR-34b-3p
           hsa-miR-34b-5p
5'- gugcucgguuuguaggcagugucauuagcugauuguacuguggugguuacaaucacuaacuccacugccaucaaaacaaggcac  -3'   exp
    (((((..((((...(((((((...((((.((((((((((.....)).))))))))))))...)))))))....))))..)))))   reads  mm  sample
    ............uaggcagugucauuagcugauug.................................................   1      0   seq
    .............aggcagugucauuagcugauug.................................................   3      0   seq
    .............aggcagugucauuagcugauugu................................................   26     0   seq
    .............aggcagugucauuagcugauugua...............................................   3      0   seq
    .................................................caaucacuaacuccacugccauc.............   1      0   seq
    ..................................................aaucacuaacuccacugccauc.............   1      0   seq
```

Cell line : NHOST differentiated
miRBase precursor : hsa-mir-34b
Total read count : 35
hsa-miR-34b-3p read count : 2
hsa-miR-34b-5p read count : 33
remaining reads : 0

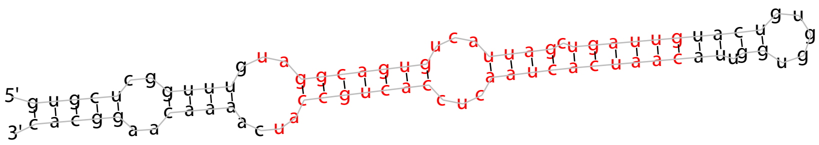

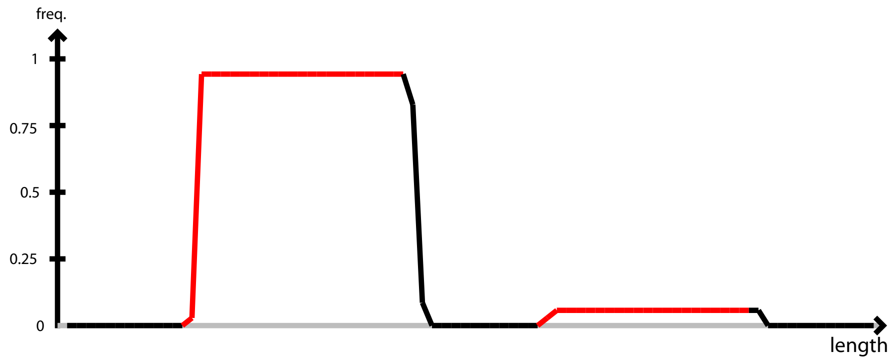


```
                           hsa-miR-34b-3p
         hsa-miR-34b-5p
5'-  gugcucgguuuguaggcagugucauuagcugauuguacugugguggguuacaaucacuaacuccacugccaucaaaacaaggcac  -3'   exp
     ((((..((((...((((((...((((.((((((((((.....)).)))))))))))...)))))))....))))..)))))            reads   mm   sample
     ............uaggcagugucauuagcugauug.................................................  1       0    seq
     ............aggcagugucauuagcugauug..................................................  3       0    seq
     ............aggcagugucauuagcugauugu.................................................  26      0    seq
     ............aggcagugucauuagcugauugua................................................  3       0    seq
     .................................................caaucacuaacuccacugccauc............  1       0    seq
     ..................................................aaucacuaacuccacugccauc............  1       0    seq
```

Supplementary data S6: miRDeep analysis of miR-34b sequencing data for IOR-MOS and differentiated NHOST cells.

The plot shows the percentage of reads for each individual base in the mature miRNA. The annotated sequences are given in red, and the identified sequences and their number of reads are listed below.

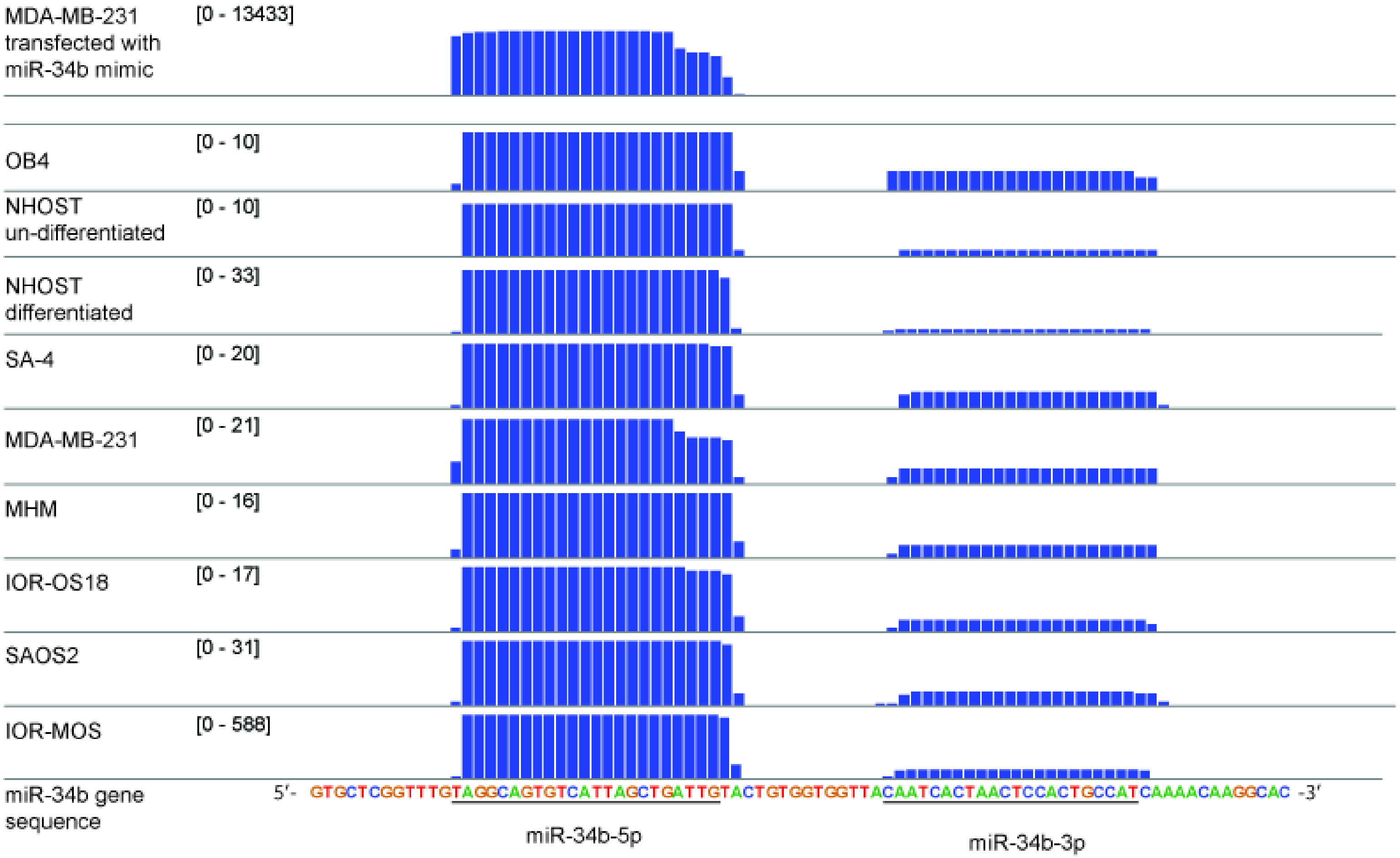

Supplementary data S7: Raw sequencing reads of mature miR-34b from both non-malignant and malignant cells.
Bars indicating the percentage of reads for the individual bases in the miR-34b gene sequence, obtained from raw sequencing data of MDA-MB-231 and noncancerous mesenchymal stromal cells (NHOST) before and after osteogenic differentiation, osteoblast primary culture (OB4), and liposarcoma (SA-4) and osteosarcoma cell lines (MHM, IOR-OS18, SAOS2, IOR-MOS). The annotated sequence is underscored. The numbers in brackets represent the number of total reads for each cell line.
